# Biofilm Accelerates As(III) Oxidation on Reactive MnOx Coated Filter Sand in Groundwater Filters

**DOI:** 10.1021/acsestwater.5c01031

**Published:** 2025-12-01

**Authors:** Roos Goedhart, Emiel Kruisdijk, Doris van Halem

**Affiliations:** Water Management Department, Faculty of Civil Engineering and Geosciences, Delft University of Technology, Stevinweg 1, 2628 CN, Delft, The Netherlands

**Keywords:** Arsenic, Biofilters, Surface-Catalytic Oxidation, Biological Oxidation, Manganese Oxides, Rapid Sand Filtration

## Abstract

Removal of carcinogenic arsenic (As) from groundwater is essential for providing safe drinking water. Arsenate (As­(V)) is more effectively removed in groundwater filters than arsenite (As­(III)), making the oxidation of As­(III) to As­(V) a key step in the treatment process. This study distinguishes between surface-catalytic and biological As­(III) oxidation on natural manganese oxide (MnO_*x*_) coated filter sand, since it is unknown which pathway dominates in filters. The MnO_*x*_ coated sand was collected from a full-scale groundwater filter and consisted of a mixture of different abiotically and biologically formed Mn oxides, such as Birnessite and Todorokite. A lab-scale filter setup was operated with As­(III)-containing water. Within 3 weeks, a shift from surface-catalytic to biological As­(III) oxidation was observed. Initially, surface-catalytic As­(III) oxidation (*k*_CHEM_ = 0.318 min^–1^) was coupled to Mn­(II) release at a ratio of 0.96, approximating the stoichiometric ratio of 1. This coupling disappeared over time, indicating the biological nature of the reaction, as confirmed by microbial inhibition. An increase in relative abundance of the known As-oxidizing families *Comamonadaceae*, with *Polaromonas* as the dominant genus, and *Microscillaceae* were found post experiments. Except for these changes, the microbial community on the sand grains stayed relatively similar prior to and post experiments. No significant changes in the physical-chemical properties of the MnO_*x*_ coating were found post experiments. A first-order biological As­(III) oxidation rate constant *k*_BIO_ of 4.64 min^–1^ was found, yielding a half-life of 9 s. This represents a 14-fold acceleration compared with surface-catalytic oxidation, revealing that kinetic limitations rather than surface passivation can be attributed to the loss of surface-catalytic oxidation. Our study demonstrates that biological oxidation of As­(III) can outpace the acknowledged oxidizing power of MnO_*x*_, offering a potential new pathway for the development of effective As removal systems.

## Graphical abstract

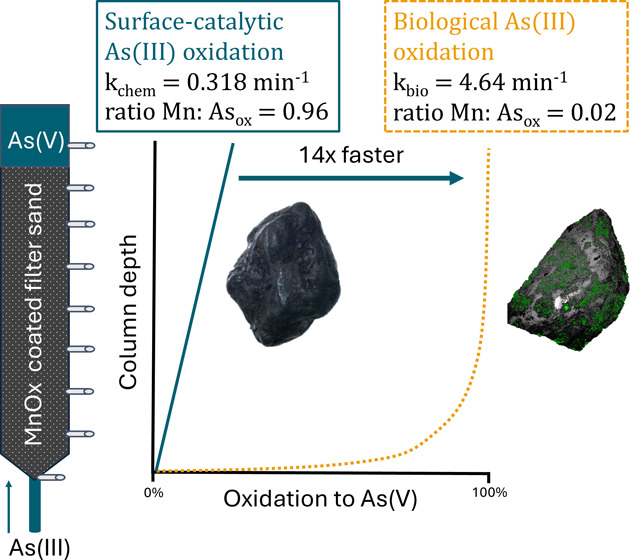

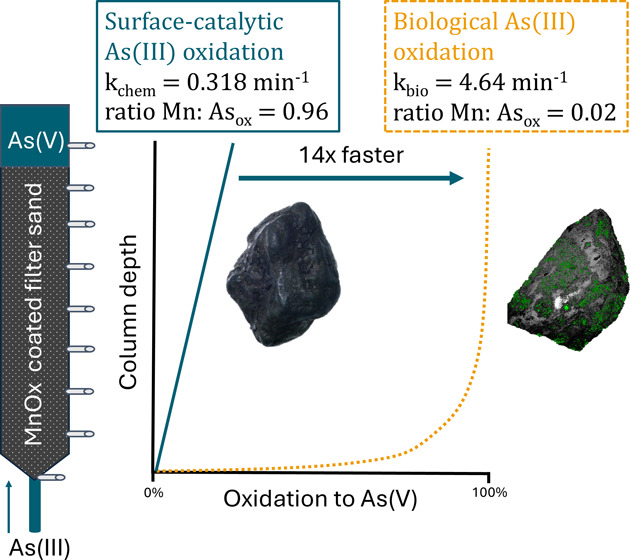

## 1 Introduction

Elevated concentrations of arsenic (As) have been detected in groundwater in various countries, including Bangladesh, Vietnam, Argentina, the Unites States of America, and India.^1^ An estimated 94 to 220 million people are potentially exposed to As concentrations above 10 μg/L in groundwater, from which the majority (94%) lives in Asia.^2^ Groundwater serves as a common source for drinking water, and As, known for its carcinogenic properties, should be removed to provide safe drinking water. This remains a challenge due to lack of acceptable, affordable, robust, and sustainable arsenic-safe water alternatives and treatment methods.^3^

The most common treatment method for groundwater is aeration followed by rapid sand filtration (RSF), primarily designed to target the removal of iron (Fe^2+^), manganese (Mn^2+^), and ammonium (NH_4_^+^). Removal of As in the treatment chain takes place through adsorption onto Fe oxides. The efficiencies of As removal vary strongly per location, caused by different Fe/As ratio’s present and co-occurrence of competing anions (e.g., phosphate).^4^ Several field studies report levels of >50 μg As/L in the treated water,^5–7^ which can be toxic at lifelong consumption.^1^ More modern technologies such as membrane filtration or anion exchange can also be ineffective in removing As.^8,9^ In anaerobic groundwater, with typically near-neutral pH, As occurs predominantly as As­(III) in the form of H_3_AsO_3_ (p*K*_1_ = 9.2). Under oxidizing conditions the pentavalent As­(V) dominates in the form of HAsO_4_^2–^ or H_2_AsO_4_^–^ (p*K*_a_ = 2.2, p*K*_b_ = 6.9 respectively).^10^ Fe oxides typically have a higher adsorption capacity for As­(V), underlining the importance of oxidizing As­(III) to As­(V) in the treatment process.^11^

Aeration alone is insufficient to oxidize As (half-life of 4–9 days^12^) within the time scale typical for RSF (residence time approximately 10 min). However, in aerobic full-scale filters, As­(III) oxidation has been observed to occur within minutes, indicating the crucial role of the filter bed in this oxidation process.^13^ The acceleration of As­(III) oxidation has been attributed to three possible mechanisms: 1) generation of reactive oxygen species (ROS) during oxidation of Fe­(II)^14^ 2) surface-catalytic oxidation by Mn oxides on the filter coating^15–18^ or 3) biological oxidation by arsenic oxidizing bacteria (AsOB).^19–21^ The current research studies As­(III) oxidation on Mn oxide-coated filter sand, examining the contribution of the surface-catalytic and biological mechanism. It is currently unknown which pathway dominates in filters.

The reactive Mn oxides coating on sand grains forms through oxidation of dissolved Mn­(II) present in the influent water, facilitated by manganese oxidizing bacteria (MnOB).^22^ The formed amorphous biogenic Mn­(III/IV)-oxides (called MnO_*x*_ hereafter) acts as strong oxidants that can further enhance surface-catalytic Mn­(II) oxidation^23,24^ and As­(III) oxidation.^17^ The coating can also harbor a biofilm, thereby supporting biological oxidation. Figure 1 provides a schematic overview of the relevant As­(III) oxidation processes on the MnO_*x*_ coated sand.

**Figure 1:**
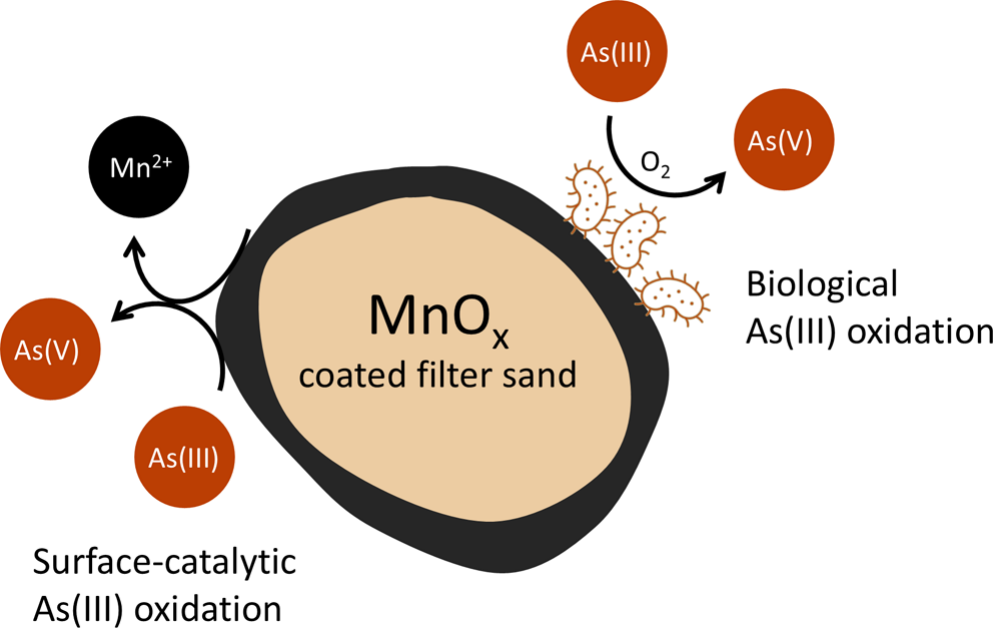
Schematic overview of surface-catalytic and biological As­(III) oxidation on MnO_*x*_ coated filter sand.

During surface-catalytic oxidation, the Mn­(III/IV)-oxide coating serves as the electron acceptor, releasing Mn­(II) while oxidizing As­(III) at a stoichiometric ratio of 1 (eq 1). Scott and Morgan (1995) showed that synthetic MnO_2_ is able to oxidize As­(III) with a half-life of 10–20 min, which is also reported for MnO_*x*_ powder scraped from a mature filter sand coating.^17^ A reduction in As­(III) oxidation rates over time have been observed, caused by surface passivation of the reactive MnO_*x*_ coating.^25–27^ Other constituents in the water such as Fe and Mn can hinder As­(III) oxidation on the MnO_*x*_ surface in pH neutral water,^17^ so the availability of a MnO_*x*_ coated surface does not necessarily mean that As­(III) gets oxidized, emphasizing the complex and currently unpredictable system.

Groundwater native bacteria are able to oxidize As­(III) in biofilters,^19,28^ although only a few studies applied biological As­(III) oxidation to treat groundwater.^21,29,30^ Note that the aforementioned studies observed biological As­(III) oxidation in laboratory or pilot setups only. The reaction equation of catalyzed As­(III) oxidation by arsenic oxidizing bacteria (AsOB) is given in eq 2. Van Le et al. (2022) analyzed microbial communities within a functioning household sand filter.^31^ While evidence of biological Fe and Mn oxidation was observed, AsOB were not found. Hence, although biological oxidation of As­(III) might occur in filters, the question remains whether AsOB will thrive on a reactive and mature MnO_*x*_ filter coating.

Surface-catalytic

1 $$H_{3}{AsO}_{3}+MnO_{2}+2H^{+}\to {Mn}^{2+}+H_{3}{AsO}_{4}+H_{2}O$$

Biological

2 $$H_{3}{AsO}_{3}+O_{2}+2H^{+}\overset{AsOB}{\to }H_{3}{AsO}_{4}+H_{2}O$$

Achieving complete As­(III) oxidation by the MnO_*x*_ coating eliminates the need for the addition of oxidative agents in the treatment chain. In rural areas where groundwater contains elevated concentrations of As, the addition of oxidative agents is often not an option, because of high chemical costs, maintenance difficulties, and inadequate infrastructure. Complete As­(III) oxidation by the filter bed itself can also contribute to the aim of the Dutch drinking water companies of lowering the As guideline to 1 ug/L, while maintaining a simple, robust and economically viable system.^32^

The objective of this study is to distinguish between biological and physicochemical surface-catalytic As­(III) oxidation on MnO_*x*_ coated filter sand. Identifying the oxidation mechanisms can help to determine which pathway dominates inrapid sand filters. A lab-scale filter study was performed, using MnO_*x*_ coated sand ripened in a functioning full-scale treatment plant. The kinetics of both surface-catalytic and biological As­(III) oxidation were determined by collecting a time series of As speciated height profiles before and after the addition of a microbial inhibitor.

## 2 Materials and Methods

### 2.1 Lab-Scale Filter Study

Figure 2 shows a schematic overview of the laboratory column setup. The unchlorinated tap water flowing through the column was spiked with a 15 mg/L As stock solution, derived from a 0.05 M sodium arsenite solution (Merck). The pH of the stock solution was kept below 3 by the addition of 37% HCl (Fluka). The column influent water had an average As concentration of 494.6 (STD ± 47.2) μg/L and a pH of 7.92 (STD ± 0.09). The As­(III) concentration varied between 52% and 86% of the total arsenic concentration in the influent, and the remainder was As­(V). The columns, made from transparent PE, were wrapped in aluminum foil to avoid light intrusion. A second column was attached to the outflow of the column and filled with GEH 102 adsorption media to remove residual arsenic before discharging.

**Figure 2:**
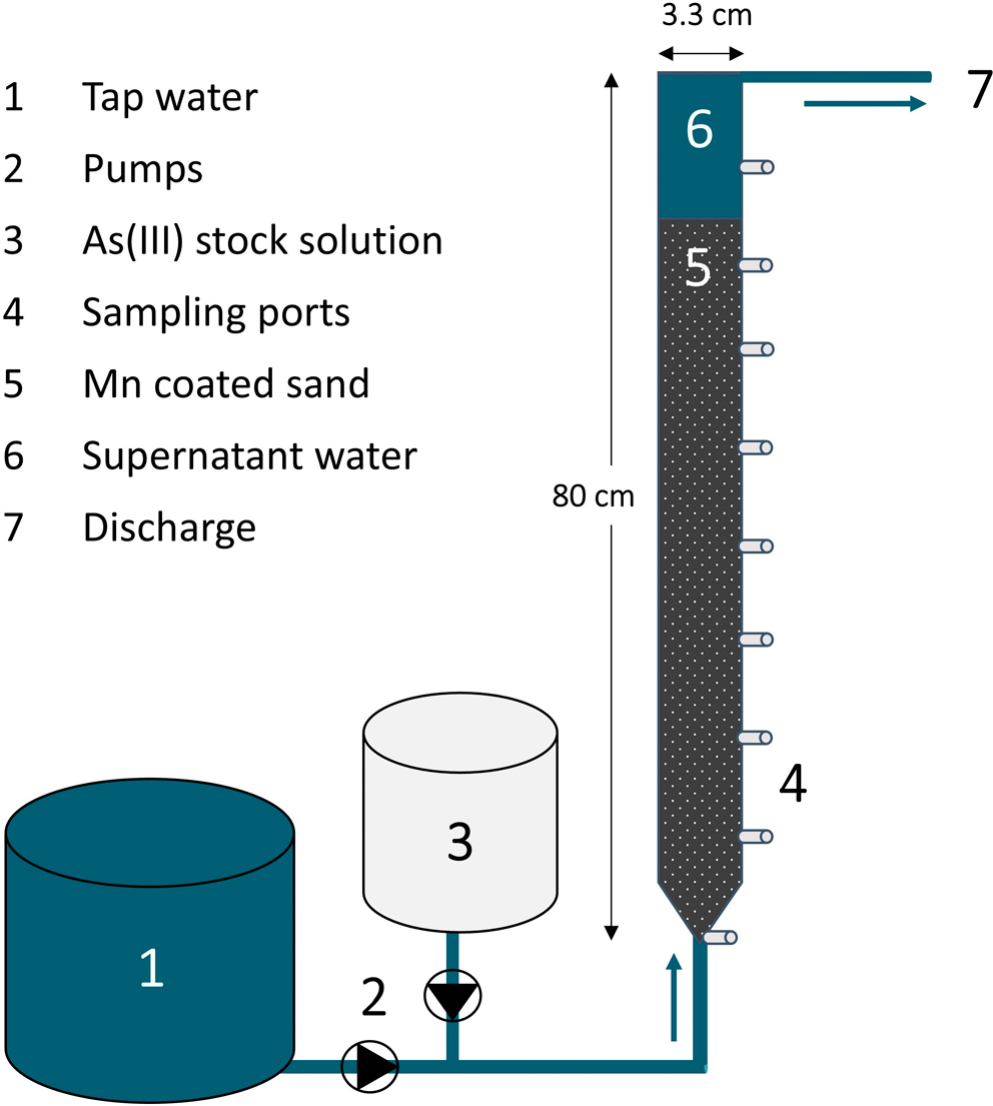
A schematic overview of the laboratory lab-scale filter setup, consisting of an upflow column filled with MnOx-coated sand collected at a full-scale groundwater filter.

The design details and operational settings of the column are given in Table 1. Compared with groundwater filters (3–10 m/h), the column operated on a relatively high flow rate of 20 m/h. The high flow rate facilitated the replacement of many pore volumes within a short duration, saturating the adsorption sites and, consequently, establishing a sorption equilibrium. From that moment on, adsorption could no longer mask the potential occurrence of As­(III) oxidation, which enabled monitoring the oxidation state of arsenic. In total, the column remained operational for 66 days, corresponding to the treatment of 11.5 m^3^ water and 41,000 pore volumes. Pore volumes are the number of times the pores are completely flushed with water. No clogging was observed during the experiment, and therefore, backwashing was not applied.

**Table 1:** Design Details and Flow Settings of the Lab-Scale Filter

**Column design**	
Filter bed height	73 cm
Supernatant water level	7 cm
Column diameter	3.3 cm
Bed volume	624 cm^3^
Porosity	0.45 -
HRT	2.2 min
Total flow rate	20 m/h

### 2.2 MnO_*x*_ Coated Sand

The MnO_*x*_ coated sand utilized in this study was collected from the secondary filter of a treatment plant treating anaerobic groundwater in Belgium (Pidpa). At the treatment location, the aim is to achieve biological iron removal in the primary filter bed by maintaining a relatively low pH (6.9) and O_2_ concentration (3.4 mg/L). Manganese is subsequently removed in the secondary filter, resulting in a clear black coating on the sand, high in Mn content. Also arsenic was present in the groundwater at this location (40 μg/L) and removed to ±0.5 μg/L in the primary filter. The aeration step after the primary filter raised the O_2_ concentration to 6.7 mg/L and the pH to 8.4. In the secondary filter, the Mn concentration of 34 μg/L lowered to <0.5 μg/L. For further details on the water quality data at this treatment location, see Supplementary Table 1.

The composition of the coating of the sand is determined by using different methods. The coating was extracted as proposed by Claff et al. (2010).^33^ This extraction method allows for the determination of the Mn, Fe and As concentrations, by dissolving the coating and using inductively coupled plasma mass spectrometry (ICP-MS, Analytikal Jena PlasmaQuant MS). Additionally, the coating’s properties were investigated using digital light microscope (VHX-5000, Keyence), Environmental SEM (Quanta FEG 650: FEI at 0.5 °C and 6–8 mbar H_2_O atmosphere), X-ray diffraction (Bruker D8 Advance-ECO diffractometer with *Cu radiation* and Bragg–Brentano geometry), Raman spectroscopy (Renishaw Raman Invia Reflex: excitation wavelength, 514 nm; dwell time, 10 s; iterations, 10; grating, 3000 lines per mm) and EPR spectroscopy (Bruker EMX Bruker EMX plus X-band EPR spectrometer: microwave frequency, 9.797 GHz; Microwave power, 20 mW; modulation frequency, 100 kHz; modulation amplitude, 10 G; room temperature). The Brunauer–Emmett–Teller (BET) theory was used to determine the surface area of the sand grains in duplicate (Micromeritics Tristar II at 77K). Around 2 g of sand was used for analysis, pretreated at 60 °C for 15 h before measurement. The original biofilm on the sand grain prior to experiments was prepared for imaging using a Leica DM6 Stellaris 8 Confocal Scanning Laser Microscope using a 25x water immersion objective (0.95 NA, WD 2.5 mm) (RRID: SCR_026519). The biofilm was fixed by immersing the grain in a 4% paraformaldehyde solution for 90 min, followed by three washing steps in a phosphate-buffered saline (PBS) solution and a sequential 10 min immersion in ethanol solutions of increasing concentration (20%, 50% and 80%). For staining, 5 μL of a 10,000× SYBR GreenⓇ solution in DMSO (Sigma Aldirch) was dissolved to 1× in PBS. The granule was submerged in the staining solution for 30 min before microscopic analysis.

### 2.3 Water Quality Analysis

To study the filter behavior over time, height profiles were taken by sampling the influent, the effluent, and at different depths of the column (Figure 2). The samples were collected at a low velocity to minimize disturbing the system. The pH and Dissolved Oxygen (DO) were measured using SenTix 980 and FDO 925 probe, respectively (WTW, Germany). As­(III) was speciated immediately by an Amberlite IRA-400 chlorite form anion ion-exchange resin using a method similar to that described by Gude et al. (2016).^13^ Around 8 mL of resin was added to a 10 mL syringe. Initial preparation involved two washes of the resin with deionized water followed by flushing the resin with one volume of the sample itself. When the resin was empty, 10 mL of the sample was dosed and collected. The resin was discarded after treating 4 samples. The gathered samples were preserved at 4 °C until analysis.

Concentrations of As and Mn were quantified by using inductively coupled plasma mass spectrometry (ICP-MS, Analytikal Jena PlasmaQuant MS). Prior to analysis, the samples were acidified (ROTIPURAN Ultra 69%, 1% v/v) and filtered through a 0.20 μm nonsterile Millex Syringe filter with Durapore membrane.

### 2.4 DNA Extraction

DNA extraction is required to study the microbial composition of the biofilms on the sand grains. However, extracting DNA from metal-coated sand with Qiagen DNeasy Powersoil Pro Kit or Fast DNA Spin Kit for Soil, yielded DNA concentrations below 0.1 ng/μL, also after an additional sonication step. These low yields, as often reported in metal-rich environments,^35,36^ hinder downstream processes such as sequencing.

A new method was therefore developed to release the DNA from MnO_*x*_ coated sand by dissolving the coating in ammonium oxalate. A 0.5 M ammonium oxalate monohydrate (Sigma-Aldrich, ≥ 99%) solution was brought to a pH of 3 by the addition of 37% HCl (Fluka) and was subsequently sterilized by autoclaving. Five times 1 g of sand was sonicated in 0.5 mL Tris+EDTA buffer solution (Sigma-Aldrich, BioUltra) with a VialTweeter (UP200 St Hielscher) for two times 10 s (in pulses), followed by a vortex step of 2 s. A vacuum filter unit was used, holding a sterile 0.2 μm filter paper. The liquid (including detached coating) of the five vials was transferred together on the filter paper. Subsequently, 10 mL of the ammonium oxalate solution was added, which dissolved the black manganese coating. The filter paper was subsequently flushed with 5 mL of DNA-free water (VWR). The filter paper was transferred to the PowerBead Pro Tube of the QIAamp powerfecal pro DNA kit (Qiagen) and the manufacturer’s instructions were followed further onward. The concentration of extracted DNA was quantified using Qubit 4 Fluorometer and Qubit dsDNA HS assay kit (Invitrogen, Waltham, MA, USA) following the manufacturer’s protocol. Sequencing of the extracted DNA was carried out by Novogene Europe. The methods are given in the Supporting Information.

### 2.5 Sodium Azide Addition

After 36 days of operation (22,400 pore volumes), the microbial activity of the column was suppressed with sodium azide (NaN_3_), since NaN_3_ has no effect on the physicochemical properties of the MnO_*x*_ coated filter material.^37^ NaN_3_ was added to the As-spiked tap water, with a final concentration of 25 mM NaN_3_. The column was immersed in this solution for a duration of 12 h. Subsequently, the column was flushed with 15 L of the NaN_3_ As solution at a flow rate of 2.5 m/h. Just before and after the inhibition, 2 g of grains were removed from the bottom of the column to determine the concentration of ATP (Luminultra, DSA test kit). After inhibition, the column was returned to its original operational settings. After 2 days, corresponding to 670 pore volumes, a height profile was taken again to assess the impact of the NaN_3_ dosing on the system. The system remained operational for 30 more days.

### 2.6 Simulation As and Mn Concentrations

Based on the As­(III) concentrations measured, a first-order rate constant (*k*) was determined via:

3 $$As\left(III\right)={As\left(III\right)}_{in}\times e^{-kt}$$

The simulated As­(V) and Mn­(II) concentrations were calculated via the surface-catalytic reaction equation as given in eq 1, meaning that oxidizing 1 mol As­(III) forms 1 mol of As­(V) and releases 1 mol Mn­(II). As­(tot) was determined by summing the simulated As­(V) and As­(III) concentrations.

## 3 Results

### 3.1 As­(III) Oxidation on MnO_*x*_ Coated Sand

Figure 3 shows the As­(III) and As­(V) concentrations in the effluent of the column for the first 36 operational days, corresponding to 22,400 pore volumes. The mean As influent concentrations are indicated by the blue and yellow dashed lines, corresponding to 305.9 (STD + 27.7) and 188.7 (STD + 52.0) μg/L for As­(III) and As­(V), respectively. The system remained oxic (DO > 6.6 mg/L) over time. The average pH of the influent and effluent water was 7.92 (STD ± 0.087) and 7.86 (STD ± 0.054) respectively.

**Figure 3:**
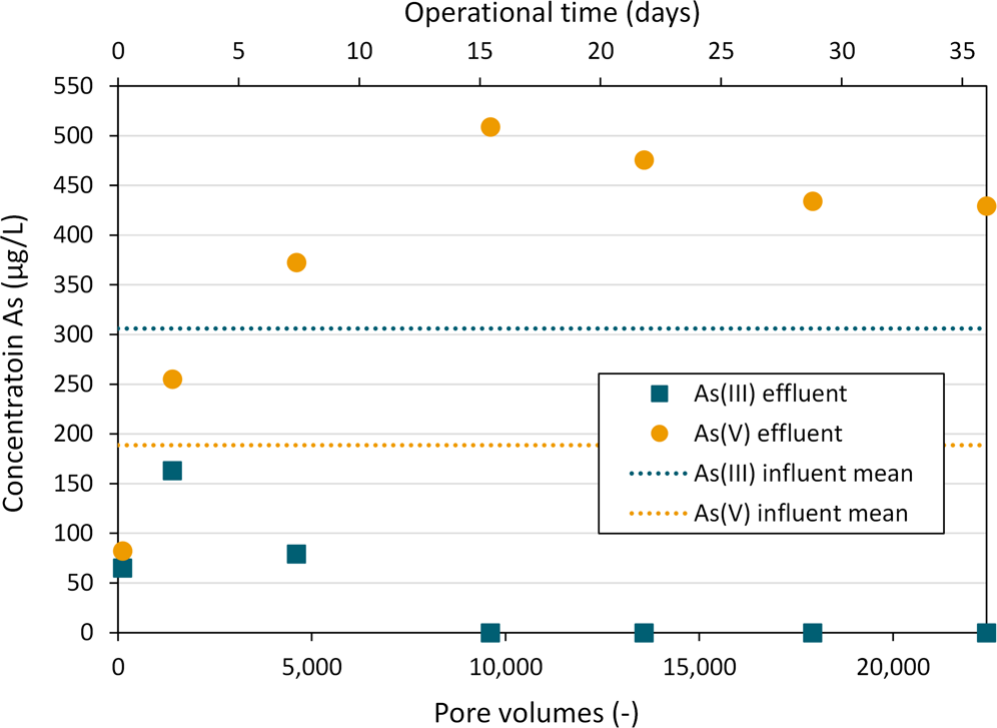
As­(III) and As­(V) effluent concentrations of the columns over time (days) and over the corresponding replacement of pore volumes (−). The dotted lines are the mean As­(III) (top) and As­(V) (bottom) influent concentrations. Adsorption of arsenic was 75% on day 1, 16% on day 3 and <6% on the remaining days.

On day 1, both As­(III) and As­(V) were found to adsorb in the column, resulting in a total As removal of 440.6 μg/L (75%). This loss of total As in the column due to adsorption may mask the occurrence of oxidation mechanisms. On day 3, 16% of total As was removed, which further decreased to below 6% for subsequent data points. Production of As­(V) by oxidation of As­(III) was observed starting from day 3 (1,400 pore volumes), corresponding to an effluent As­(V) concentration exceeding the As­(V) influent concentration. The fraction of As­(V) in the effluent further increased over time, and on day 16 (9,600 pore volumes) As­(III) was no longer detected in the effluent. In other words, a fully oxidizing system was reached in which all incoming As­(III) was recovered in the effluent as As­(V).

### 3.2 Oxidation over the Height of the Filter Bed

Figure 4 shows the As­(tot), As­(III), As­(V) and Mn­(II) concentration profiles over the height of the column for day 3, marking the first day of observable As­(III) oxidation (Figure 3), and for day 29, which is close to the end of the experiment. The height profiles of the other days can be found in Supplementary Figure 1. Total As removal was 82.1 (16%) and 4.8 μg/L (1.1%) on day 3 and day 29, respectively. This illustrates that although a fraction of As was still adsorbed in the column, the majority could be recovered in the effluent (84–99%).

**Figure 4:**
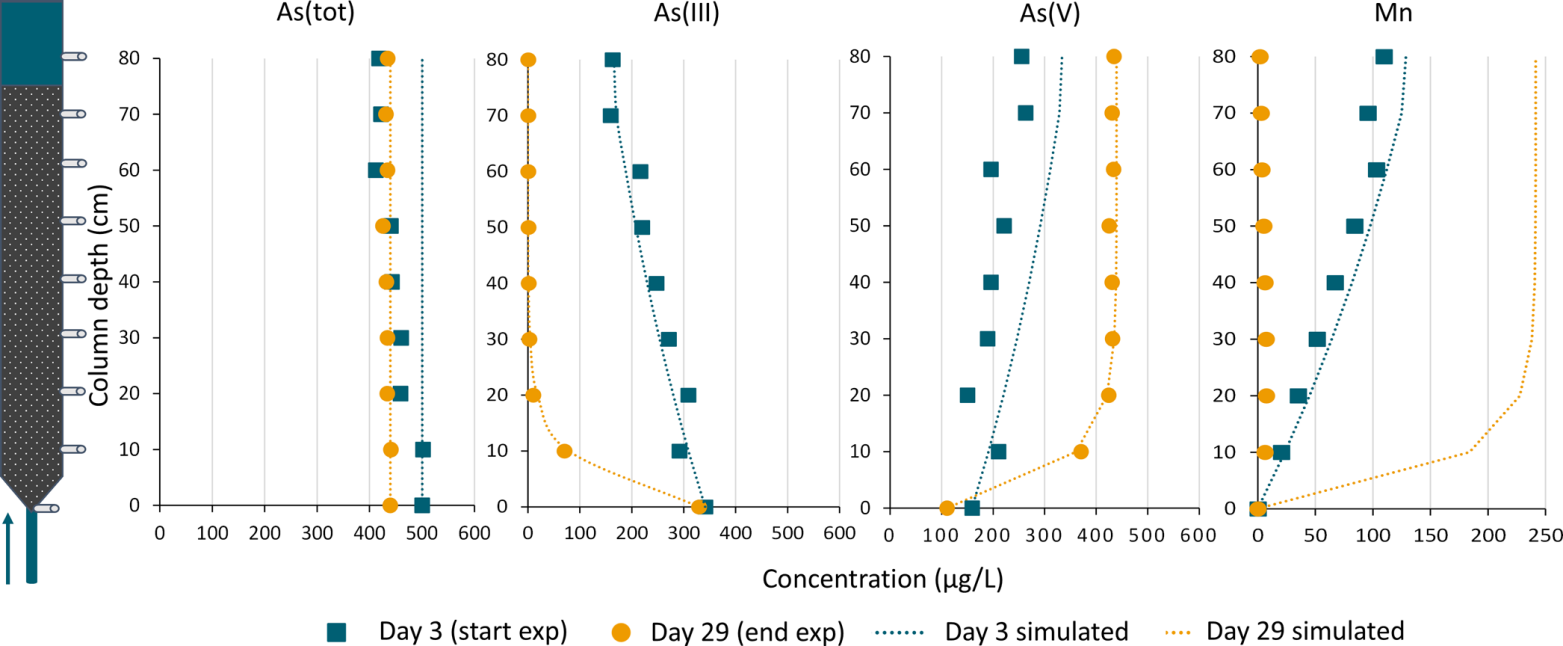
Height profiles of As­(tot), As­(III), As­(V) and Mn­(II) at the start (day 3, blue squares) and the end (day 29, yellow circles) of the experiment. The simulated concentrations with first-order kinetics are also depicted (dotted lines).

On day 3, the As­(III) concentration decreased over the height of the column from 342 to 163 μg/L, of which 95 μg/L was identified as As­(V) in the effluent (Figure 4). The simulated concentrations for day 3 are also shown in Figure 4: a first-order As­(III) oxidation rate was found (*R* ^2^ = 0.9291) with a constant *k*_As(III)_ of 0.318 min^–1^. The As­(tot) and As­(V) concentration measured were slightly lower than predicted by the simulation based on the As­(III) concentration, likely because of the 16% adsorption of As onto the column. Assuming this oxidation is surface-catalytic, the simulated Mn­(II) released based on *k*_As(III)_ and a Mn:As­(III) ratio of 1 predicted a Mn­(III) concentration of 129 μg/L Mn. The Mn­(II) concentration measured in the effluent was 110 μg/L.

On day 29, As­(III) concentrations were below the detection limit (6 μg/L) after the first 30 cm in the column. The first-order reaction constant increased to a *k*_As(III)_ of 4.64 min^–1^ (*R* ^2^ = 0.9750). At this rate, the simulated surface-catalytic release of Mn­(II) should reach 241 μg/L in the effluent; however, the concentration of Mn­(II) observed was below the detection limit (6 μg/L).

### 3.3 Characteristics of Mineral-Microbe Coating

The MnO_*x*_ coating of the sand appears homogeneously black by the eye and under the digital light microscope (Figure 5A). The confocal microscope and SEM images (Figure 5B and Supplementary Figure 2) reveal a more fractured morphology with both rough and smoother patches. The surface area found using the BET theory was 1.51 (±0.01) m^2^/g, which is in a similar order of magnitude as found by Arora et al. (2025).^38^ Figure 5B shows that the biofilm grows in patches on the MnO_*x*_ coated sand grain. The surface morphology of the sand directly collected from the sand filters is similar to that of the sand exposed to As in the columns (Supplementary Figure 2). Table 2 provides an overview of the contribution of Mn (15.3%), Fe (5.9%), and As (0.0032%) in the coating, measured by chemical extraction. The relatively high levels of Fe in the coating contrast with the initial homogeneous impression of the coating. Additionally, the large deviation in Fe suggests that the amount of Fe precipitates varied greatly among grains. The presence of Fe in the coating was confirmed by point EDS measurements (Supplementary Table 2), which additionally showed the presence of carbon and oxygen, as well as calcium and silica.

**Figure 5:**
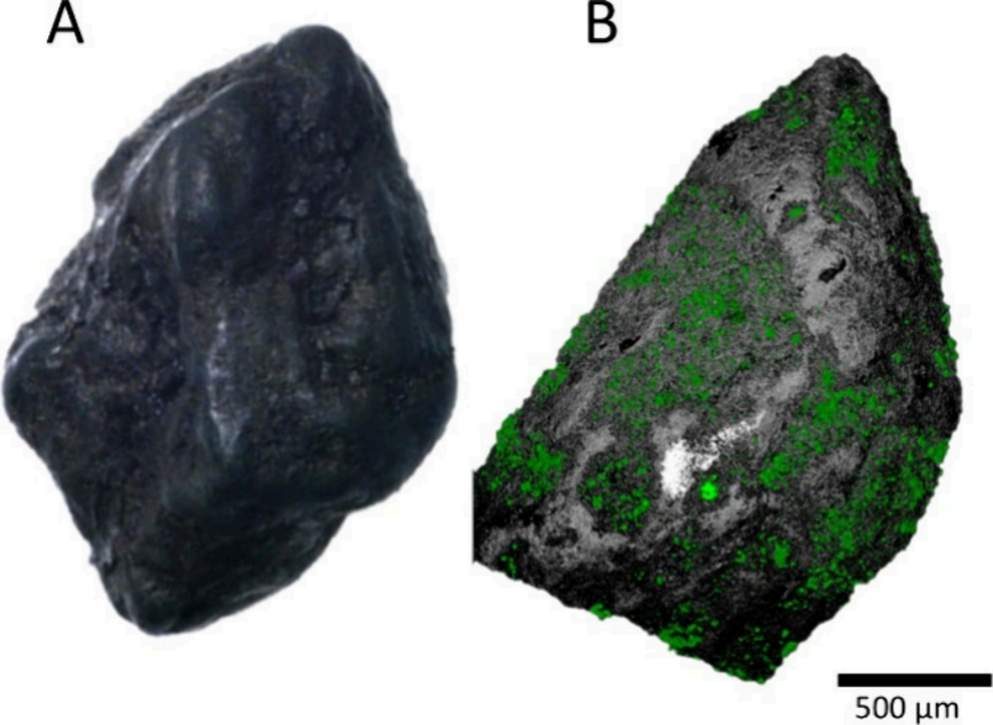
Representative digital light (A) and confocal (B) microscopy images of a sand grain prior to the experiments. In (B): green = SYBR GreenⓇ stained microbes, gray = reflection of the sediment

**Table 2:** Mn, Fe, and As Content in the Coating of the Sand Prior to Experiments, Measured by Chemical Extraction^a^

**Element**	Average (mg/g)	SD (mg/g)	Average (%)
Mn	153.4	24.96	15.3
Fe	58.6	30.79	5.9
As	0.03254	0.01537	0.0032

a *n* = 3, SD = standard deviation.

Powder X-ray diffraction (XRD) was performed on the grain coatings prior to and post experiments. In both coatings, the Mn mineral Todorokite was detected (Supplementary Figure 3). In the spectra of the coating post experiments, slightly higher crystallinity was found, including crystallized Birnessite. Other typical Fe or Mn precipitates formed in groundwater filters, such as poorly ordered ferrihydrite or amorphous Birnessite, cannot be detected by using XRD.

The Raman spectra of the sand prior and post experiments are shown in Figure 6A and 6B respectively. Different regions of the same samples were measured at different angles. The spectra are dominated by the signal of the Quartz sand. All spectra show a peak around 625 cm^–1^, which is likely a signal from the Mn oxides. Some spectra, both prior and post experiments, have an additional peak around 570 cm^–1^. The variation in spectra among the different angles suggests that the coating is a mixture of different Mn oxides. The single peak around 625 cm^–1^ could be attributed to either Todorokite or Ranciéite.^39^ Birnessite is known to have two Raman peaks between 575 and 585 cm^–1^ and 625–646 cm^–1^,^40^ which could explain the second peak at 570 cm^–1^. The slightly lower wavenumbers could be caused by strain effects.

**Figure 6:**
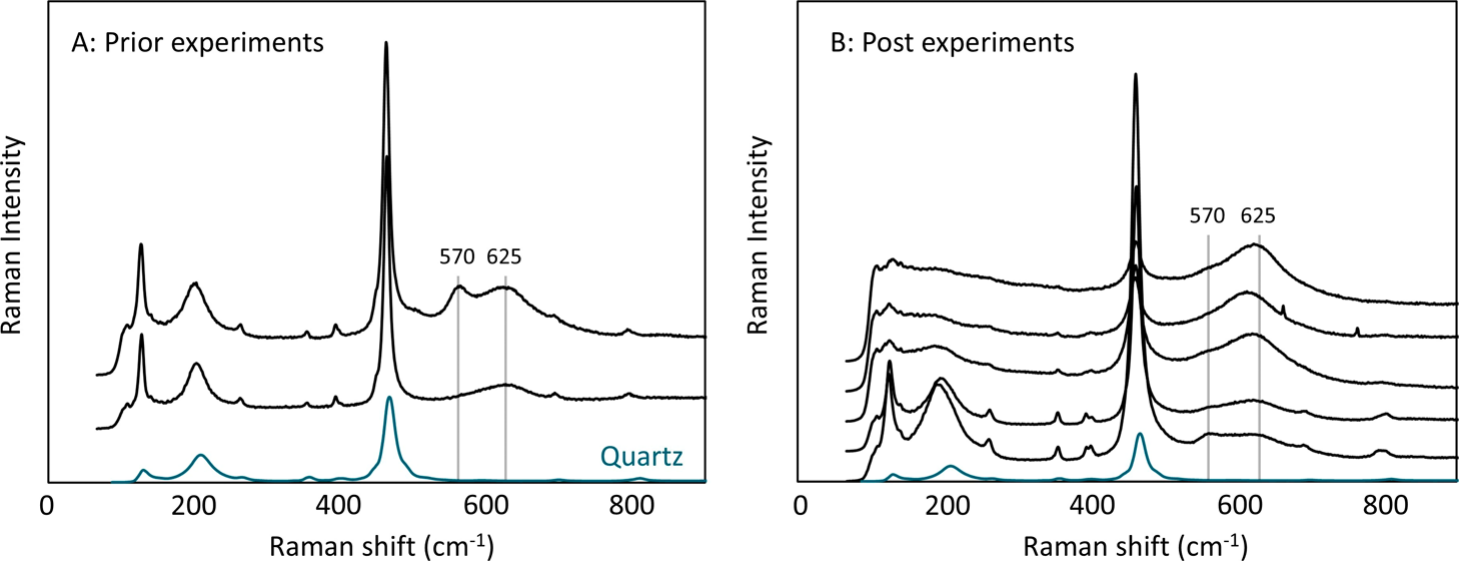
Raman spectra of Mn coated sand prior (A) and post (B) experiments. The different spectra represent different measurements of different regions and angles of the same sample. The bottom spectrum (blue) is a reference spectrum of Quartz. The peaks likely caused by the MnO_*x*_ coating are labeled (570 and 625 cm^–1^).

Samples were measured by EPR to study the biotic or abiotic origin of the manganese oxides (Figure 7). The spectra are dominated by a very broad signal of circa Δ2000 G and a sharp peak of Δ180 G around 2400. In both spectra, a small signal near 1600 G was found, which is consistent with mononuclear Fe^3+^.

**Figure 7:**
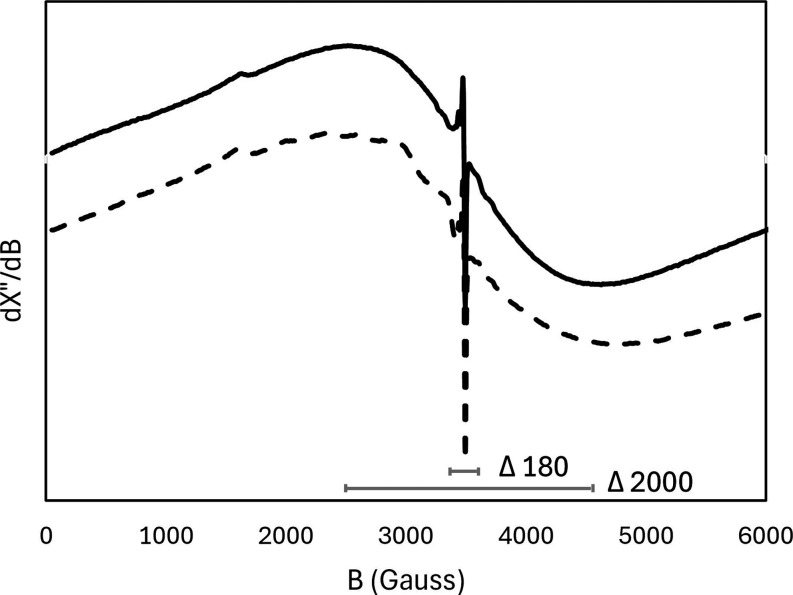
EPR Spectra of sand prior (top, solid) and post (bottom, dash) experiments. The differences in Gauss of the spectra are given.

### 3.4 Microbial Community on Mineral-Microbe Coating

The microbial community on MnO_*x*_ coated sand grains prior and post experiments are given in Figure 8. The communities are presented in relative abundance (RA) in the percentage of OTUs at the taxonomic ranks of class and family. At the class level, no major shift in the microbial community was observed when comparing prior and post experiment samples. Across all samples, *Gammaproteobacteria*, *Alphaproteobacteria*, and *Nitrospiria* were the most abundant classes with RAs ranging from 22–28%, 24–35%, and 22–27%, respectively. At the family level, *Nitrospiraceae* dominated all samples, but shifts were observed from prior to post experiment. *Comamonadaceae* emerged post experiments, reaching a RA of 17% in both samples. Within the family *Comamonadaceae*, *Polaromonas* was the main detected genus, with a RA of 12% in both samples post experiment (data not shown). Additionally, the level of the family *Microscillaceae* increased to 4% and 2% in the duplicate samples (data not shown). *Comamonadaceae, Polaromonas* and *Microscillaceae* are known arsenic oxidizing bacteria, as shown by e.g. Crognale et al. (2019),^19^ Huang et al. (2024),^41^ Osborne et al. (2013),^43^ Roy et al. (2021)^42^ and Su et al. (2022).^44^ Post experiment, the growth of *Pseudonocardiaceae* was also observed until a RA of 5–6%; this family has previously been found in As-rich environments.^50,51^ *Gallionellaceae* was no longer detected post experiments.

**Figure 8:**
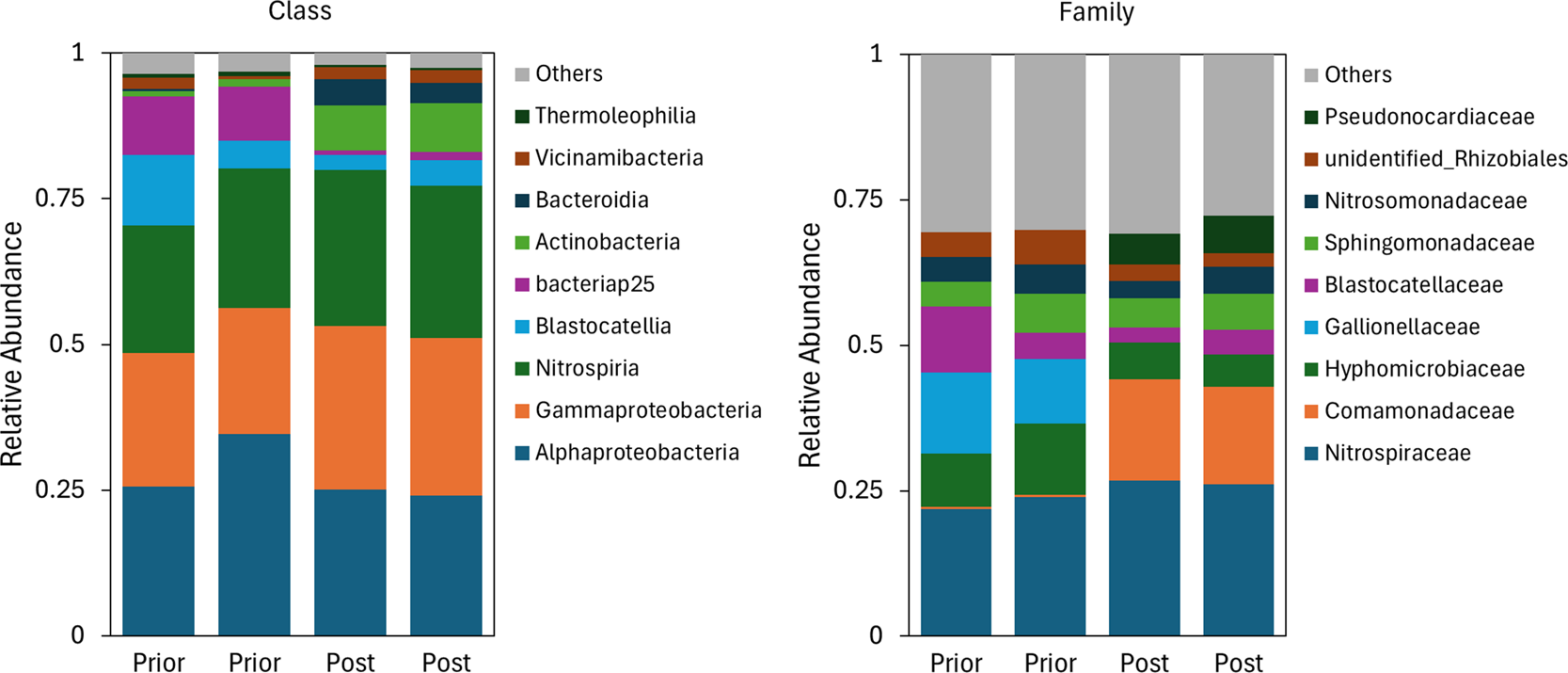
Distribution histogram of relative abundance of taxonomic rank class (left) and Family (right) of the microbial community on the sand grains prior and post experiments. Both samples in duplo.

The addition of NaN_3_ to the columns did not cause major shifts in the community composition compared to post experiment (Supplementary Figure 4). It is important to note that the figures represent relative abundances, which do not reflect absolute microbial concentrations.

### 3.5 Ratio Mn­(II) Released over As­(III) Oxidized

Figure 9A presents the Mn­(II) concentrations in the effluent of the column during the experiment. In addition, the molar Mn­(II):As­(III) ratio is shown for released Mn­(II) over oxidized As­(III). At the start, the released Mn­(II) (110 μg/L) aligned with the stoichiometry of the surface-catalytic As­(III) oxidation reaction by the reduction of Mn oxides (eq 1). However, the molar ratio of 1 was temporary, because the ratio gradually decreased to 0.28 and 0.07 on day 8 and 16, respectively. By day 22, or 13,600 pore volumes, the Mn­(II):As­(III) ratio had reached 0, as Mn­(II) effluent concentrations were below the detection limit (6 μg/L). A ratio of 0 is expected during biological As­(III) oxidation, as given in eq 2.

**Figure 9:**
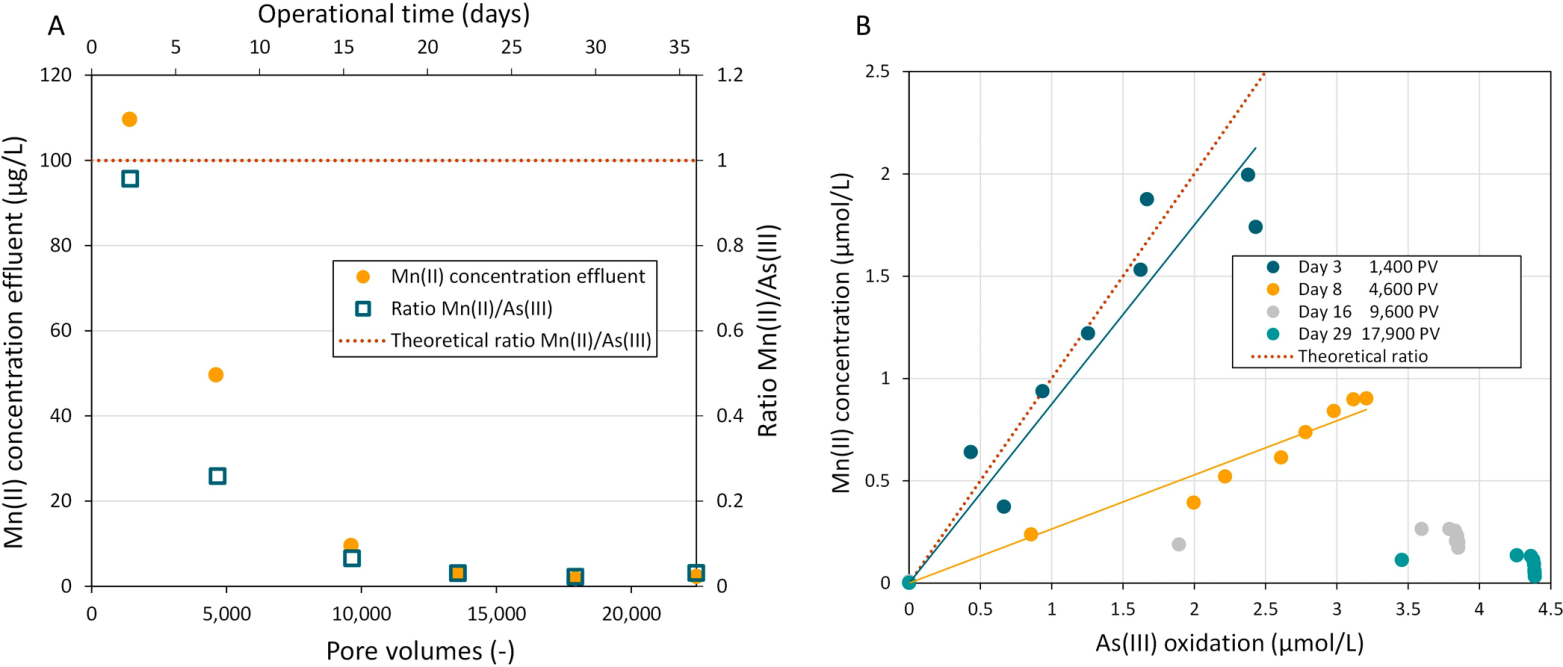
A) Mn­(II) concentration in the effluent of the column and the ratio of Mn­(II) released over As­(III) oxidized for different days and the corresponding replacement of pore volumes (−). B) As­(III) oxidized over the Mn­(II) concentration released at the different tap heights on day 3, 8, 16, and 29 (PV = pore volumes). The theoretical line depicts 1 mol of Mn­(II) release per mole of As­(III) oxidized according to eq 1.

To investigate the Mn:As­(III) ratio over the height of the filter column, the fraction of oxidized As­(III) versus the Mn­(II) concentration for the different taps in the column is depicted in Figure 9B. Particularly on days 3 and 8, a clear linear trend is visible (*R* ^2^ 0.97, 0.99), with the ratio being closest to the stoichiometric value of 1 for day 3. For the later days of the experiment, the relationship between Mn­(II) release and As­(III) oxidation becomes less apparent. The As­(III) oxidation increased over these days, while the amount of released Mn­(II) decreased over time. The ratios found confirm that the pathway of As­(III) oxidation is driven by Mn oxide reduction at the start of the experiment, but moves toward biological As­(III) oxidation over time.

### 3.6 Inhibition of Biological As­(III) Oxidation

Figure 10A shows the As­(V) and As­(III) concentrations in the effluent before and after microbial inhibition by NaN_3_ on day 36. Prior to inhibition, a completely oxidizing system was obtained, as all incoming As­(III) was converted to As­(V) in the first 30 cm (*k*_As(III)_ = 4.64 min^–1^). After exposure to NaN_3_, As­(III) oxidation dropped by approximately 65%, with >200 μg/L As­(III) in the effluent after inhibition (*k*_As(III)_ = 0.198 min^–1^). The addition of NaN_3_ decreased the ATP concentration by 2 orders of magnitude (16 × 10^4^ to 66 × 10^2^ pg of ATP/g of sand).

**Figure 10:**
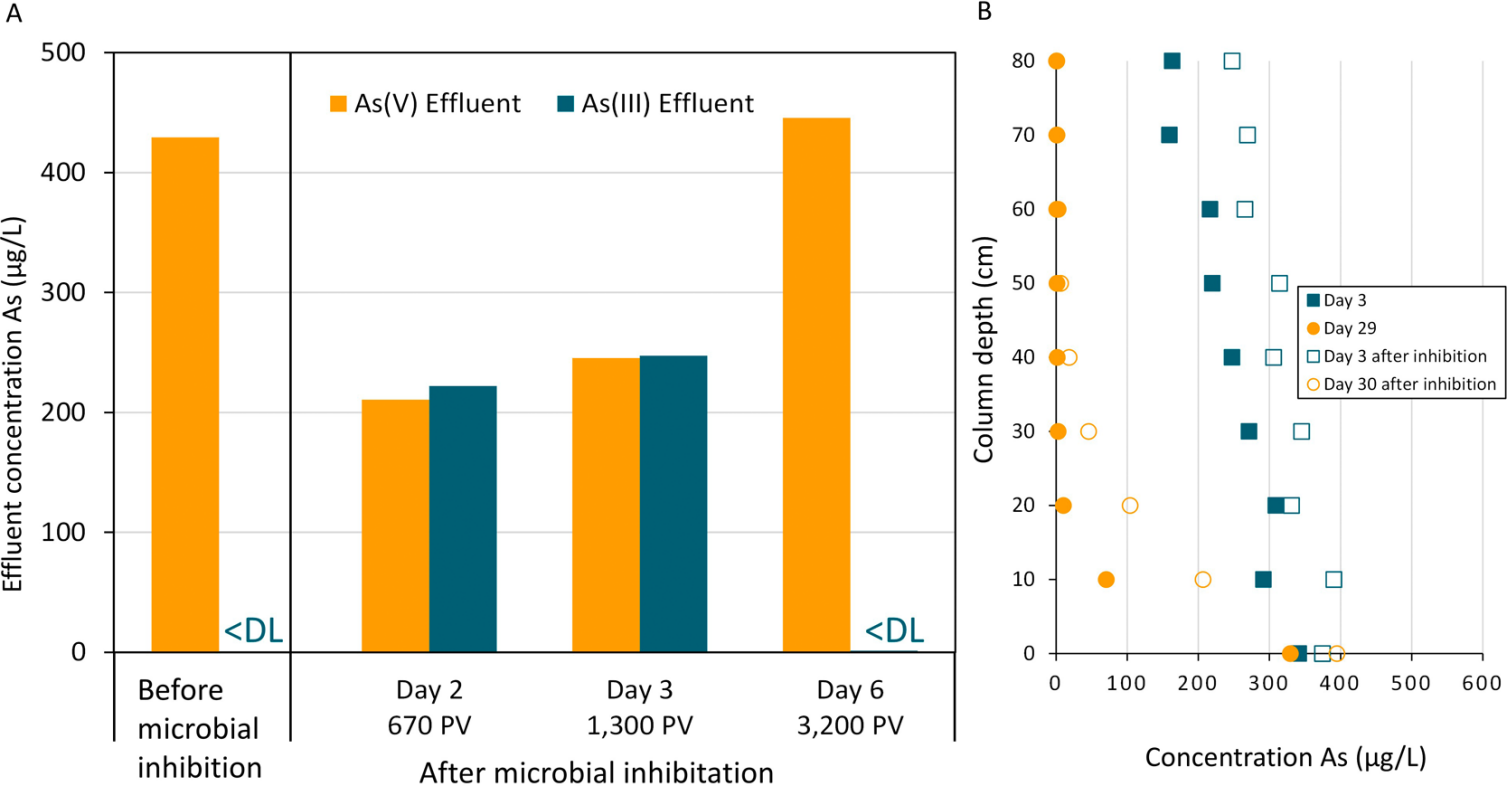
A) As­(V) and As­(III) effluent concentrations before and after microbial inhibition by NaN_3_. < DL = below detection limit. PV = pore volumes. B) Height profile of As­(III) depletion on day 3 and day 29 before and after microbial inhibition by NaN_3_.

This inhibition demonstrates that the arsenic oxidizing bacteria found on the MnO_*x*_ coating were responsible for the observed acceleration of As­(III) oxidation in the period prior to inhibition. After exposure to NaN_3_, these organisms could not oxidize As­(III) for several days. On day 6 after inhibition, complete As­(III) oxidation was again observed in the column yet at a rate slower than prior to inhibition (*k*_As(III)_= 1.55 min^–1^).

The height profiles for As­(III) oxidation, either before or after microbial inhibition, on similar operational days show a rather striking resemblance (Figure 10B). On day 3, *k*_As(III)_ was 0.318 and 0.198 before and after inhibition, respectively. At the end of the experiment, on day 29, *k*_As(III)_ had reached 4.65 and 2.48 min^–1^, before and after inhibition, respectively. This indicates that biological As­(III) oxidation developed again within days after inhibition. The rate constants for the other experimental days are presented in Supplementary Figure 5.

## 4 Discussion

### 4.1 Shift from Surface-Catalytic to Biological As­(III) Oxidation on MnO_*x*_ Coated Sand

At the start of the experiment (day 3 or 1,400 pore volumes), As­(III) oxidation was coupled to the reduction of Mn oxides, with an Mn­(II):As­(III) ratio of 0.96, closely resembling the stoichiometric ratio of 1. This shows that oxidation was chemical according to the reaction formula for surface-catalytic As­(III) oxidation (eq 1). The column performed uniformly over the height of the filter bed with similar ratios observed at every tap height (Figure 9B). By day 22 (13,600 pore volumes), Mn release dropped to below detection limit (6 μg/L), while a completely oxidizing As­(III) system was obtained (Figure 9A). As­(III) oxidation without Mn release indicates that the reaction became biological, based on the reaction formula of eq 2. Additionally, known AsOB *Comamonadaceae*, *Polaromonas* and *Microscillaceae* emerged in the biofilm on the MnO_*x*_ coating during experiments. Introducing NaN_3_, a microbial inhibitor, resulted in a 65% decrease in the efficiency of the As oxidizing system. This further exhibits the biological nature of oxidation at the end of the experiment.

Surface-catalytic oxidation by Mn oxides was inadequate to completely oxidize all incoming As­(III). The incomplete oxidation is likely attributed to kinetic limitations rather than surface passivation of the Mn oxides. Surface passivation of the reactive MnO_*x*_ coating would decrease the oxidation efficiency, yet in our system, biological oxidation supplanted this process before passivation could occur. This is further supported by the return to surface-catalytic oxidation after microbial inhibition, demonstrating that passivation has not occurred. Additionally, surface passivation would cause the reaction to have two different kinetic regimes as described by Nielsen-Franco and Ginder-Vogel (2023),^52^ yet such a two-phase pattern over the height of the filter column was not observed. A first-order model has been shown to describe the kinetics well in our system (Figure 4).

### 4.2 Characteristics of the Stable MnO_*x*_ Coating

Exposing the MnO_*x*_ coated sand to arsenic and the growth of an arsenic oxidizing biofilm on the coating had no major effects on the characteristics of the coating. The MnO_*x*_ coating on the sand grain is likely a mixture of different Mn oxides, in several oxidation states [Mn­(II), Mn­(III) and Mn­(IV)]. The Raman spectra showed variability over different areas of the coating, detecting possibly Todorokite, Birnessite and Ranciéite both prior to and post experiments (Figure 6). XRD also revealed the presence of Todorokite (Supplementary Figure 3) both prior and post experiments but only detected Birnessite post experiments. However, the poor crystallinity of MnO_*x*_ and the presence of amorphous iron oxides make a proper characterization of manganese oxides by XRD challenging.^39^

The manganese minerals have likely both a biotic and abiotic origin; Kim et al. (2011) concludes that in EPR a broad peak of Δ*H* > 1200 can be attributed to abiogenic Birnessite, while a sharp peak of Δ*H* < 600 is typical for biologically formed Birnessite.^53^ The spectra of both coatings prior to and after experiment show a clear broad and sharp peak (Figure 7). However, the theory of distinguishing the biological nature of manganese minerals on the line width of the narrow EPR spectra has been recently challenged.^54^ Furthermore, iron oxide minerals on sand have been shown to exhibit broad EPR spectra with some similarities of the manganese oxide signals.^55^ In both spectra, a small signal near 1600 G was found, which is consistent with mononuclear Fe^3+^.

### 4.3 Emerging AsOB in Biofilm on MnO_*x*_ Coating

No major shifts in the microbial community were observed upon exposure of the drinking water biofilm to arsenic (Figure 8). At the Family level, *Nitrospiraceae* dominated all samples post and prior experiments, despite the absence of nitrite and ammonium in the tap water supplied during the experiments (<0.01 mg/L NO_2_ and <0.05 mg/L NH_4_). *Comamonadaceae* emerged in the samples post experiment. *Comamonadaceae* contain known arsenite oxidizers and are frequently found in As rich environments.^19,41,42^ The main genus found within *Comamonadaceae* was *Polaromonas*, capable of oxidizing arsenite.^43^ Similarly, an enrichment of *Microscillaceae* was found post experiments, aligning with findings by Su et al. (2022), who reported their enrichment in arsenic contaminated rice terraces.^44^ *Gallionellaceae*, known iron oxidizers, disappeared upon exposure to As during the experiments. Apparently, *Gallionellaceae* cannot survive in the dosed water matrix, in which iron is absent and arsenic is present. The presence of *Gallionallaceae* on the sand grains prior to experiments likely originates from flush out of the primary treatment filter, where biological iron removal takes place.

Both *Comamonadaceae* and *Microscillaceae* likely played roles in the accelerated As oxidation in the columns. However, it cannot be definitively concluded that they were the only As oxidizing bacteria present. The minimal shifts in community composition after As enrichment suggest that a typical sand filter microbial community can tolerate As concentrations of 500 μg/L. Besides As, there are very little nutrients present in the tap water. However, whether the abundant families such as *Nitrospiraceae* are passively present or actively contributing to As oxidation requires further investigation. It should be noted that 16s sequencing detects also dead bacteria, although dead cells are more likely to detach from the grains and end up in the water stream.

The addition of NaN_3_ to the columns did not cause shifts in the community composition (Supplementary Figure 4). It is important to note that the figures represent relative abundances, which do not reflect absolute microbial concentrations. It can explain the faster establishment of a fully As-oxidizing system following microbial inhibition compared to the initial experimental stage. Although the activity was suppressed, the right community composition was present for the task.

Sequencing the microbial community was possible only due to the successful DNA extraction method as proposed in Section 2.4. Dissolving the Mn mineral coating by ammonium oxalate resulted in DNA concentrations of 26–83 ng/μL, while without this additional step, the concentrations found were <0.1 ng/μL. This shows that standard DNA extraction kits are not suitable for Mn-coated sand. However, these kits are commonly used in drinking water research, which raises the question if certain colonies entangled in Mn oxides are often overseen. This highlights the necessity for a reliable and standardized method to extract DNA from filter sand.

### 4.4 Biological Oxidation 14 Times Faster

The shift from a surface-catalytic system to a biological system enhanced the As­(III) oxidation rate by 14-fold. At the start of the experiment, the determined rate constant *k*_CHEM_ (chemical) was 0.318 min^–1^. By the end of the experiment, the constant increased to a k_BIO_ (biological) of 5.64 min^–1^ with a corresponding half-life of 9.0 s. The *k* values for the remaining days can be found in Supplementary Figure 5.

The rate constants of As­(III) oxidation in manganese rich environments reported in the literature are summarized in Table 3. The range for chemical oxidation rates varies greatly, influenced by factors such as pH, As:Mn ratio, other constituents present, and the type and crystallinity of the Mn oxides.^18,27,56,57^ The oxidation and mobility of As will therefore vary in different Mn rich environments, such as in soil, the deep sea, or around mines. How other groundwater constituents, such as Fe or NH_4_, influence As­(III) oxidation on MnO_*x*_ coated filter sand should be considered for future studies. Both *k*_CHEM_ and *k*_BIO_ observed in this study notably exceeded values reported in earlier studies. An explanation could be that different Mn oxides are known to have a wide range of surface properties,^58^ e.g., naturally formed Mn oxides have a much higher surface area compared to well-crystallized synthetic δ-MnO_2_.^57^

**Table 3:** Kinetic Constants for As­(III) Oxidation in Mn Containing Systems Found in Literature (For Relevant Conditions) and in This Study

**As(III) oxidation process**	**Description exp.**	**Kinetic constant (min** ^**–1**^ **)**	**ref**
**Chemical**			
Oxidation by dissolved oxygen	Batch exp. As: 46–62 μg/L	2.2 × 10^–4^	^12^
	pH 7.6–8.5		
	Fe: 100–1130 μg/L, Mn: 9–16 μg/L in groundwater.		
Synthetic Birnessite	Batch exp. As: 7.5 mg/L	0.035	^18^
	pH 6.8 Mn:As = 12.4		
Synthetic Birnessite	Batch exp. As: 100 mg/L	0.00445	^56^
	pH 7.0 Mn:As = 12.3		
*k*_CHEM_ day 3		0.318	This study
*k*_CHEM_ day 3 after inhibition		0.198	This study
**Biological**			
Mature biologically formed Mn oxides	Batch. As: 1.2 mg/L.	0.068	^27^
	Mn:As = 64		
As(III) oxidation during biological Mn(II) oxidation	Column study. flow 7 m/h	0.23	^57^
	As: 35–42 μg/L Mn: 400–500 μg/L		
As(III) oxidation in a biological Mn(II) removal filter column	Column study. flow 7 m/h	0.56	^59^
	As: 1986 ug/L. Mn: 4.1 mg/L		
*k*_BIO_ day 29		4.64	This study
*k*_BIO_ day 29 after inhibition		2.48	This study

The biological constant *k*_BIO_ is 8 times higher compared to the highest reported value thus far in a manganese rich environment.^59^ One possible explanation for this disparity could be the presence of Mn in the influent in the study of Yang et al. (2015) as it has been found that the presence of Mn can hinder As­(III) oxidation.^17^ Another explanation might be the notably higher flow rate (20 m/h) in our study, although it has also been argued that a higher flow rate might limit As­(III) oxidation kinetics.^59,60^ Kruisdijk et al. (2024a) found a similar kinetic constant for biological As­(III) oxidation (2.94 min^–1^) in an iron oxide-coated sand filter,^61^ which indicates that the composition of the mineral coating might be of relatively little influence to the oxidation kinetics. However, the influence of the mineral coating on the oxidation efficiency requires further investigation.

Micro-organisms clearly played a vital role in the acceleration of As­(III) oxidation in the experiment. This presents a potential new pathway for the development of effective As removal systems. Poor removal of As is often attributed to the slow oxidation of As­(III) to As­(V), subsequently hampering the removal on Fe hydroxides.^62^ Practically promoting fast biological As­(III) oxidation in existing water treatment plants could potentially overcome this problem; to trigger As removal, As­(V) should form before all Fe­(II) is oxidized in the system. This finding marks a paradigm shift: in filters, Mn oxides have traditionally been demonstrated to be powerful oxidizers of As­(III),^15,16,18^ or oxidation was attributed to homogeneous oxidation by reactive oxygen species,^14^ but now it is demonstrated that biological oxidation might well dominate in filters.

## 5 Conclusion

The presented research distinguished the contribution of surface-catalytic and biological As­(III) oxidation on reactive MnO_*x*_ coated filter sand. Within 3 weeks, a shift from surface-catalytic to biological As­(III) oxidation was observed. Initially, surface-catalytic As­(III) oxidation (*k*_chem_ = 0.318 min^–1^) dominated, coupled to Mn release at a ratio of 0.96 (approximating the stoichiometric ratio of 1). This coupling disappeared over time, indicating the biological nature of the reaction as confirmed by microbial inhibition. Exposure to As during experiments led to an increase in RA of *Comamonadaceae,* with *Polaromonas* as the dominant genus, and *Microscillaceae*, which are known arsenic oxidizing bacteria. A first-order As­(III) oxidation rate constant *k*_BIO_ of 4.64 min^–1^ was found, yielding a half-life of 9 s. This represents a 14-fold acceleration, revealing that kinetic limitations rather than surface passivation can be attributed to the loss of surface-catalytic oxidation. Our study demonstrated that biological oxidation of As­(III) can outpace the acknowledged oxidizing power of MnO_*x*_, offering a potential new pathway to design effective biological As­(III) oxidation and removal filters without the need for complex technologies or the addition of oxidative chemicals.

## Supplementary Material



## Notes

Water quality data at different steps in the treatment process in Mol, Belgium; 16s Sequencing; Height profiles of all days for As­(tot), As­(III), As­(V) and Mn before and after microbial inhibition with NaN_3_; SEM Pictures of the sand prior to and post experiments; Point EDS measurement on MnO_*x*_ coated filter sand harvested from a mature filter bed; XRD spectra of sand grains coating prior and post experiments; Distribution histogram of relative abundance of taxonomic rank Class and Family of the microbial community on the sand grains prior & post experiments and after the addition of the microbial inhibitor NaN_3_; Rate constants (1/s) of As­(III) oxidation for each day before and after microbial inhibition (at day 36) and their corresponding pore volumes (PDF)
