# Supplementary material for: Biofilm Accelerates As(III) Oxidation on Reactive MnOx Coated Filter Sand in Groundwater Filters
Source: ACS ES T Water. 2025 Dec 1;5(12):7536–47. doi: 10.1021/acsestwater.5c01031 (PMC12706773; doi:10.1021/acsestwater.5c01031)
Supplement: Supplementary file 1 [file ew5c01031_si_001.pdf]

Supplementary information for

Biofilm accelerates As(III) oxidation on reactive MnOx coated filter sand in groundwater filters

Roos Goedhart[a*], Emiel Kruisdijk[a], Doris van Halem[a]

[a] Water Management Department, Faculty of Civil Engineering and Geosciences, Delft University of Technology, Stevinweg 1, 2628 CN, Delft, The Netherlands

*corresponding author (r.c.goedhart@tudelft.nl / roosgoedhart@hotmail.com)

Table 1: Water quality data at different steps in the treatment process in Mol, Belgium (Pidpa)

		Raw Influent	After Aeration	Effluent Primary filter & Aeration	Effluent Secondary Filter
pH	-	6.80	6.92	8.44	8.14
O_2	mg/L	0.15	3.40	6.71	5.87
CH_4	µg/L	21.85	13.94	1.50	
As	µg/L	40.11	40.09	0.50	0.49
Ca	mg/L		19.08	19.92	19.07
Fe	mg/L	15.25		0.095	0.0075
Mn	µg/L	33.75	33.99	33.58	0.19
NH_4	mg/L	0.40		0.238	0.003
NO_2	mg/L			0.001	0.000
NO_3	mg/L			0.47	1.19
P	µg/L	513		4.77	3.54

16s Sequencing

Sequencing of the extracted DNA was carried out by Novogene Europe. For quality control of the samples, 1.0 µg DNA per sample was used. Sequencing libraries were generated using NEBNext® DNA Library Prep Kit (manufacturer's protocol) and indices were added to the samples. The genomic DNA was randomly fragmented to a size of 350bp by shearing, subsequently DNA fragments were end polished, A-tailed, and ligated with the NEBNext adapter for Illumina sequencing, and PCR enriched by P5 and indexed P7 oligos. Purification of the PCR products was done by AMPure XP system. The formed libraries were analyzed for size distribution by Agilent 2100 Bioanalyzer and quantified using qPCR.

To generate amplicons, the V3-V4 region of the bacterial 16s rRNA gene was amplified using the primers 341 (5′-CCTAYGGGRBGCASCAG-3′) and 806R (5′-GGACTACNNGGGTATCTAAT-3′), each with an added barcode for sample identification. All PCR reactions were performed with 15 µL of Phusion High - Fidelity PCR Master Mix; 0.2 µM of forward and reverse primers, and about 10 ng template DNA. Thermal cycling consisted of initial denaturation at 98℃ for 1 min, followed by 30 cycles of denaturation at 98℃ for 10 s, annealing at 50℃ for 30 s, and elongation at 72℃ for 30 s and 72℃ for 5 min. Purification of the PCR products was done by magnetic bead purification. Samples were thoroughly mixed in equal density ratios, determined by the concentration of PCR products. The PCR products were subsequently detected and target bands were recovered. Sequencing libraries were generated with index adapters, quantified using Qubit and qPCR, and assessed for size distribution with a bioanalyzer. Illumina platform was used to pool and sequence the quantified libraries, based on effective library concentration and the required data output.

Paired-end reads were merged using FLASH (v1.2.11) (Magoc T et al.,2011) and quality-filtered using fastp (v0.23.1) (Bokulich et al., 2013). Chimera sequences were detected and removed using the UCHIME algorithm and vsearch (v2.16.0) (Edgar et al., 2011). Amplicon Sequence Variants (ASVs) were inferred using the DADA2 module in QIIME2. Taxonomic classification was performed using the SILVA database (v1381.1). The top taxa at class and family level were selected to generate relative abundance plots.

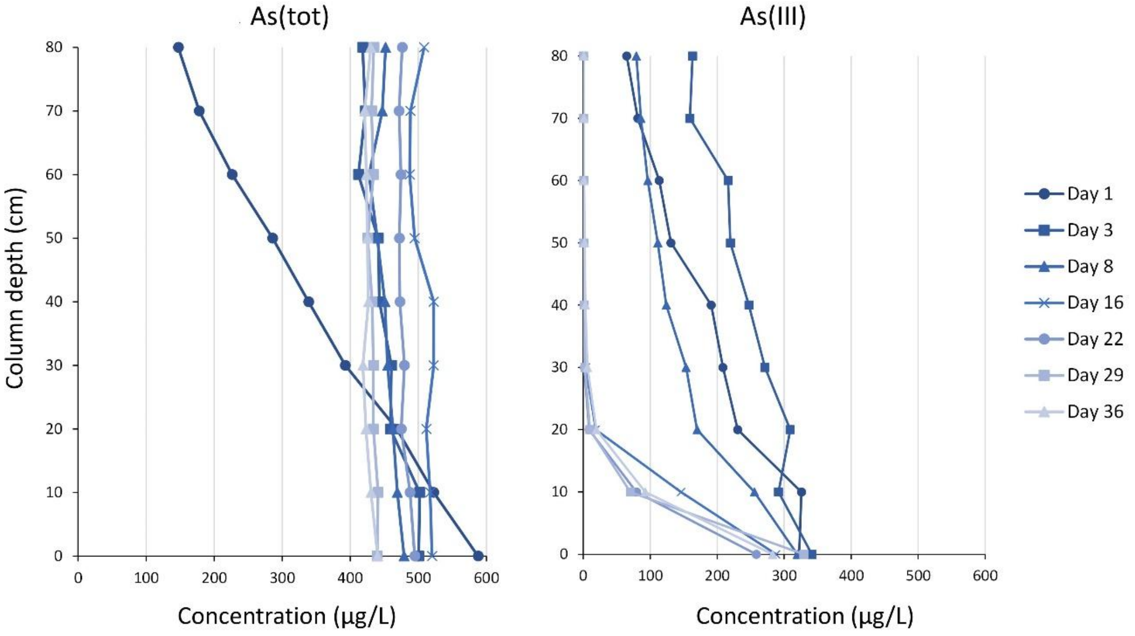

As(tot)
As(III)
Column depth (cm)
Concentration (µg/L)
Concentration (µg/L)
0
10
20
30
40
50
60
70
80
0
100
200
300
400
500
600
Day 1
Day 3
Day 8
Day 16
Day 22
Day 29
Day 36

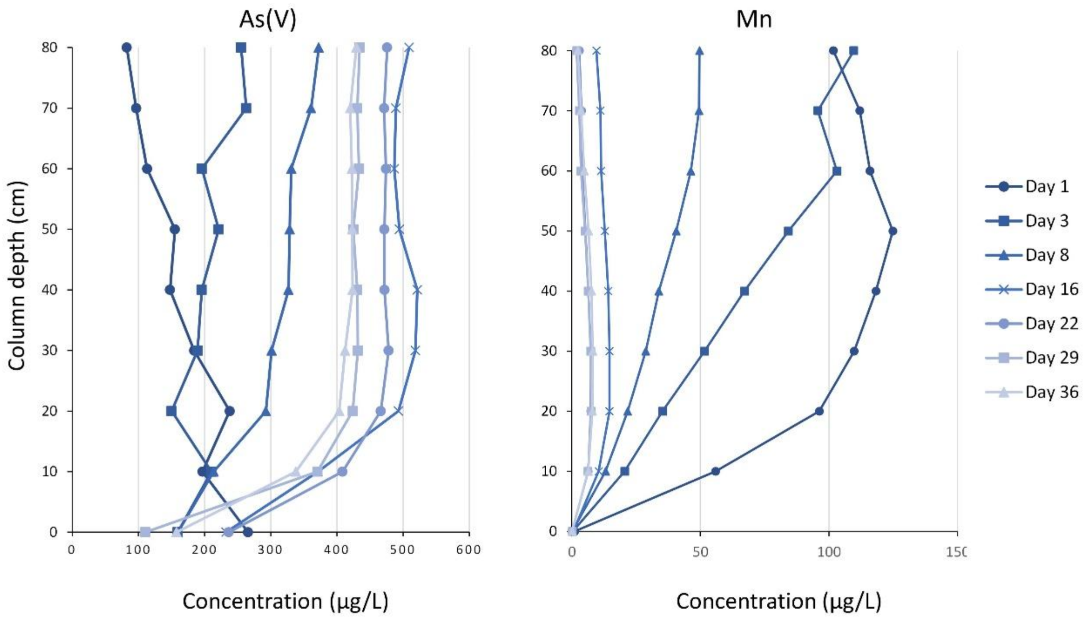

As(V)
Mn
Column depth (cm)
Concentration (µg/L)
Concentration (µg/L)
0
10
20
30
40
50
60
70
80
0
100
200
300
400
500
600
0
50
100
Day 1
Day 3
Day 8
Day 16
Day 22
Day 29
Day 36

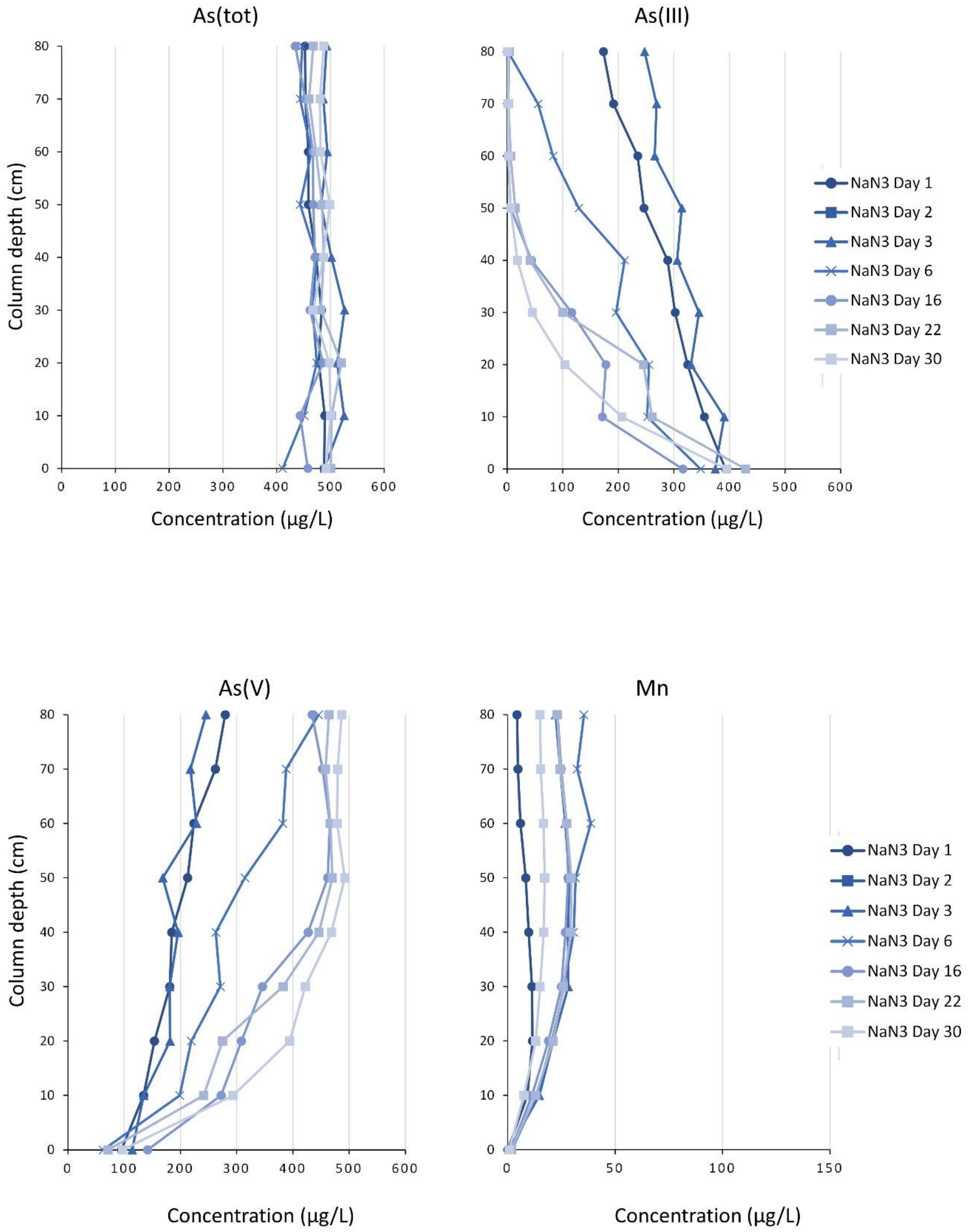

Figure 1 (two pages): Height profiles of all days for As(tot), As(III), As(V) and Mn before and after microbial inhibition with NaN_3

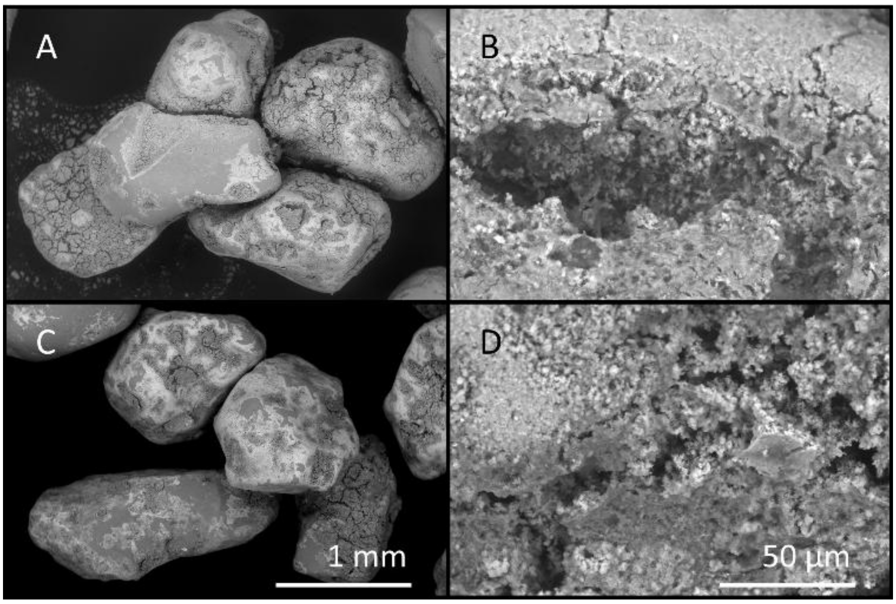

Figure 2: SEM Pictures of the sand prior to (A,B) and post experiments (C,D).

Table 2: Point EDS measurement on MnO_x coated filter sand harvested from a mature filter bed. Measured by Environmental SEM at 0.5 °C and 6 – 8 mbar H_2O atmosphere (Quanta FEG 650, FEI).

Element	Weight %	Error	Atom %	Error	Compound %
**C**	12.8	± 0.08	25.24	± 0.33	12.8
**O**	31.62	± 0.19	46.82	± 0.57	31.62
**Mg**	0.43	± 0.03	0.42	± 0.06	0.43
**Al**	0.5	± 0.03	0.44	± 0.06	0.5
**Si**	7.45	± 0.05	6.29	± 0.09	7.45
**P**	0.39	± 0.02	0.29	± 0.03	0.39
**S**	0.18	± 0.02	0.13	± 0.02	0.18
**Cl**	0.13	± 0.02	0.08	± 0.02	0.13
**K**	0.17	± 0.02	0.1	± 0.02	0.17
**Ca**	3.06	± 0.06	1.81	± 0.07	3.06
**Mn**	19.05	± 0.25	8.21	± 0.21	19.05
**Fe**	23.78	± 0.29	10.09	± 0.25	23.78
**Ba**	0.46	± 0.08	0.08	± 0.03	0.46
**Total**	100		100		100

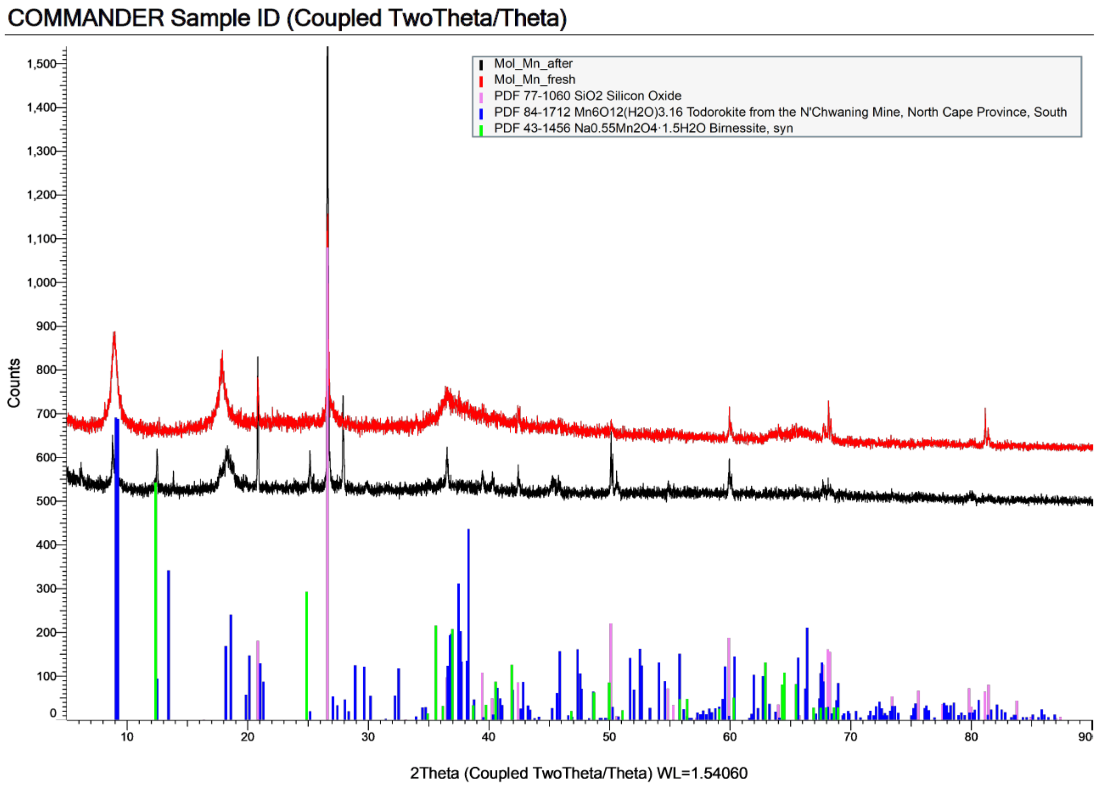

Figure 3: XRD spectra of sand grains coating prior (fresh) and post (after) experiments

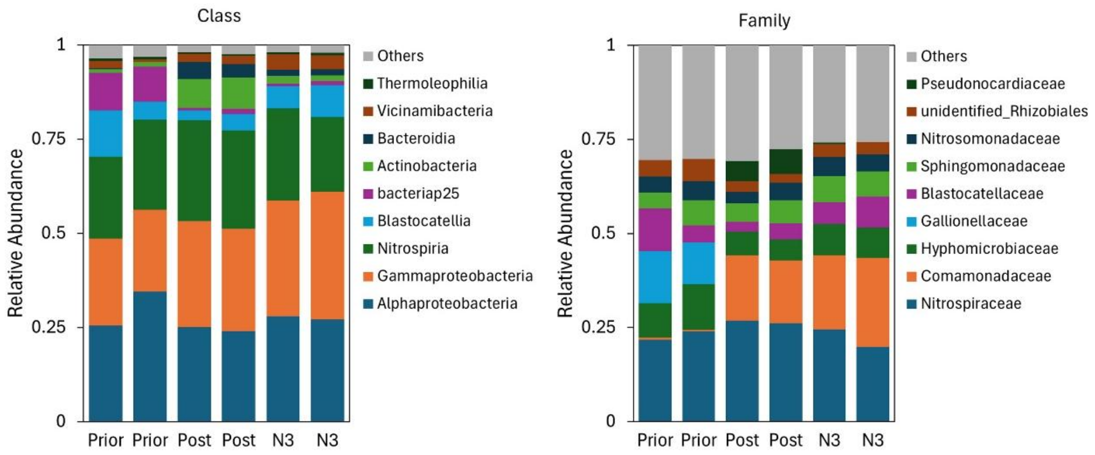

Figure 4: Distribution histogram of relative abundance of taxonomic rank Class (left) and Family (right) of the microbial community on the sand grains prior & post experiments and after the addition of the microbial inhibitor NaN_3. All samples in duplo.

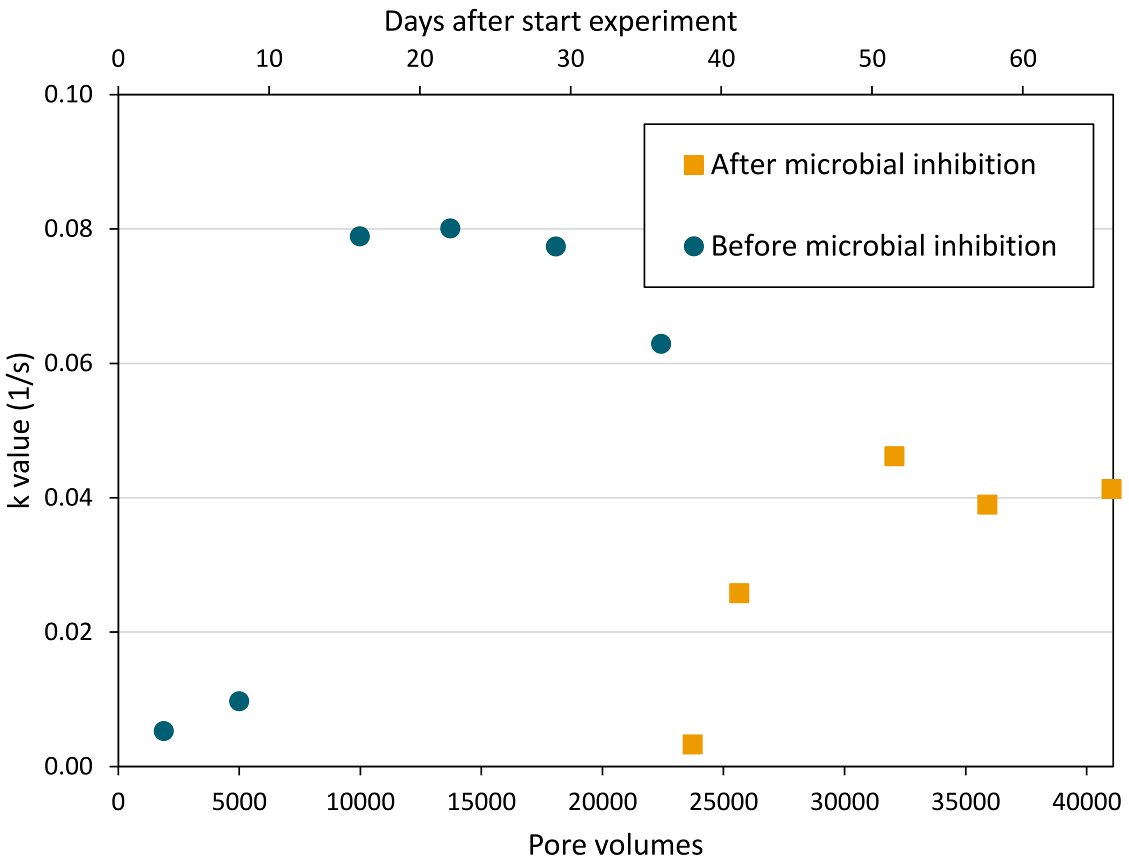

Figure 5: Rate constants (1/s) of As(III) oxidation for each day before and after microbial inhibition (at day 36) and their corresponding pore volumes.
